# Promising Application of Automated Liquid Culture System and Arbuscular Mycorrhizal Fungi for Large-Scale Micropropagation of Red Dragon Fruit

**DOI:** 10.3390/plants12051037

**Published:** 2023-02-24

**Authors:** Yaser Hassan Dewir, Muhammad M. Habib, Ahmed Ali Alaizari, Jahangir A. Malik, Ali Mohsen Al-Ali, AbdulAziz A. Al-Qarawi, Mona S. Alwahibi

**Affiliations:** 1 Plant Production Department, College of Food and Agriculture Sciences, King Saud University, Riyadh 11451, Saudi Arabia; 2 Department of Horticulture, Faculty of Agriculture, Kafrelsheikh University, Kafr El-Sheikh 33516, Egypt; 3 Department of Botany and Microbiology, College of Science, King Saud University, Riyadh 11495, Saudi Arabia

**Keywords:** areole activation, acclimatization, Cactaceae, hardening, mycorrhization

## Abstract

Red dragon fruit (*Hylocereus polyrhizus*) is an economic and promising fruit crop in arid and semi-arid regions with water shortage. An automated liquid culture system using bioreactors is a potential tool for micropropagation and large-scale production. In this study, axillary cladode multiplication of *H. polyrhizus* was assessed using cladode tips and cladode segments in gelled culture versus continuous immersion air-lift bioreactors (with or without a net). Axillary multiplication using cladode segments (6.4 cladodes per explant) was more effective than cladode tip explants (4.5 cladodes per explant) in gelled culture. Compared with gelled culture, continuous immersion bioreactors provided high axillary cladode multiplication (45.9 cladodes per explant) with a higher biomass and length of axillary cladodes. Inoculation of *H. polyrhizus* micropropagated plantlets with arbuscular mycorrhizal fungi (*Gigaspora margarita* and *Gigaspora albida*) significantly increased the vegetative growth during acclimatization. These findings will improve the large-scale propagation of dragon fruit.

## 1. Introduction

Pitayas (*Hylocereus* spp.; Cactaceae), known as dragon fruit or strawberry pear, are perennial shrubby, root-climber, frequently epiphytic plants native to tropical areas [1]. The fruits of most *Hylocereus* species are characterized by red-purple pigmented skin while the flesh color can be white, red or reddish-purple in *H. undatus, H. polyrhizus* and *H. costaricensis*, respectively [2]. The fruit is refreshing and consumed either fresh or utilized in food processing. The flowers can be cooked and eaten as a vegetable [3]. Red pulp pitaya fruits are important source of natural pigments owing to their high content of betalains [4] and play a role in the prevention of cardiovascular disease [5]. Moreover, *Hylocereus* species are greatly appreciated as ornamental pot plants or for rocky gardens. Conventional vegetative propagation of pitaya could be achieved using cladode segments/cuttings. However, it is difficult to obtain enough planting material because of the large size (~50 cm length) of the cuttings required [6], thus, a limited number of plants can be produced. Seeds can also be used to propagate *Hylocereus* species with acceptable seed germination rate, i.e., 83% for *H. undatus* [7], but this method does not guarantee true to type progenies. Additionally, it has been reported that seed-derived plants require several years to pass the juvenile phase and reach fruit production [6]. Therefore, conventional propagation using cuttings and seeds is not commercially feasible. Biotechnological tools such as plant tissue culture can be employed to improve vegetative propagation of pitaya. A year-round in vitro production of large numbers of plants in a relatively short time can be achieved through axillary bud proliferation or indirectly via callus formation (adventitious organogenesis). However, indirect shoot organogenesis does not ensure genetic fidelity of the regenerants.

Previous studies developed micropropagation in gelled media for *Hylocereus* species that include *H. undatus* [8,9,10,11], *H. costaricensis* [12,13], *H. megalanthus* [14] and *H. polyrhizus* [15,16,17]. The low multiplication rates (<10 cladodes per explant) and the considerable production costs associated with gelled cultures were major limits to the large-scale micropropagation of *Hylocereus* species. Micropropagation in gelled culture is laborious and costly compared with liquid cultures. Gelled media require small containers as compared with liquid culture systems, thus requiring more space and sub-culturing. Air-lift liquid reactor systems are pneumatically agitated reactors where the injection of gaseous substances (often air) into a specific reactor compartment provides mixing and mediated transfer of gaseous substances (i.e., O_2_ and CO_2_) with the liquid phase. The availability of nutrients and the aerated microenvironment in the bioreactor culture system provide uniform culture conditions that result in a high growth performance and improve shoot proliferation compared to the gelled culture. Additionally, renewing the nutrient medium within the same container and the intensive culture in bioreactors enable producing a large number of plants for commercial markets. However, physiological abnormalities such as hyperhydricity and foaming are associated with liquid culture systems and are plant species-dependent. The adaptation of air-lift continuous immersion and temporary immersion (ebb and flow) proved to be effective technology for mass production of plant organogenic propagules, cells and somatic embryos. A bioreactor culture system has been employed for several plant species but its application for Cactaceae plant species is limited. Recently, Bello-Bello et al. [18] reported that cladode multiplication of *H. undatus* was enhanced two-fold in a temporary immersion liquid system compared to gelled culture. To our knowledge, different liquid/bioreactor systems have not been utilized for micropropagation of other *Hylocereus* species and are yet to be explored.

In vitro plants are characterized by undeveloped photosynthetic capacity due to their heterotrophic feeding. Therefore, micropropagated plantlets suffer various stresses during their hardening and acclimatization to ex vitro conditions that reduce their growth or survival. Several strategies, such as ventilation of culture vessels, reducing sucrose supplementation and increasing light intensity, were employed to improve the plant physiological performance. The use of arbuscular mycorrhizal fungi (AMF) proved effective for the biological acclimatization of micropropagated plantlets. AMF form a symbiotic relationship with plants that ensure water and nutrient supply, promote vigorous vegetative growth and reduce mortality at this crucial stage [19,20,21,22,23]. Previous studies highlighted the significant role of mycorrhizal symbiosis in acclimatization of several micropropagated crops, i.e., *Musa* spp. [24,25,26], *Cynara cardunculus* [27] and *Gloriosa superba* [28]. Despite the potential benefits of AMF, studies on its application for in vitro propagated cacti and succulent species are scarce. Estrada-Luna and Davies Jr. [29] highlighted that AMF inoculation enhanced transplantation of *Opuntia albicarpa* micropropagated plantlets to ex vitro conditions. 

The present study aimed to employ an air-lift continuous immersion bioreactor system for the activation of areoles and cladode proliferation and to apply AMF for biological acclimatization of micropropagated *H. polyrhizus.* Overall, a significant axillary cladode proliferation was obtained in the liquid/bioreactor system compared to gelled culture. AMF significantly promoted vegetative growth and hardening of *H. polyrhizus* plantlets. These findings could be applied for the commercial micropropagation of this economically important fruit crop.

## 2. Results and Discussion

### 2.1. Effect of Explant Type and Axillary Cladode Multiplication in Gelled versus Liquid/Bioreactor Culture System

**Figure 1:**
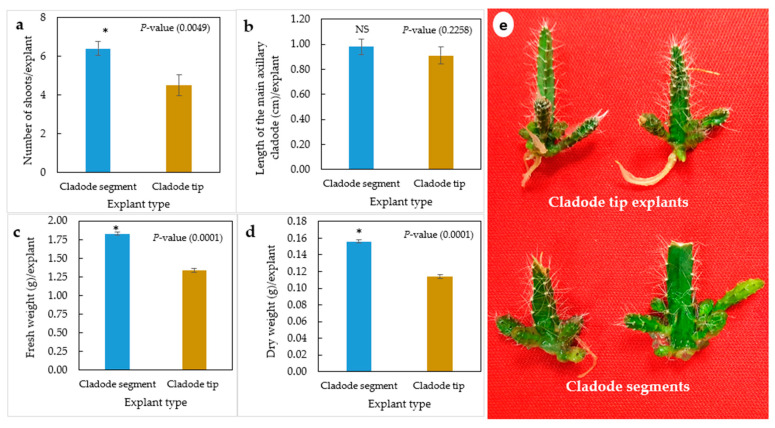
Effect of explant type (cladode segment and cladode tip explants) on multiplication of *H. polyrhizus* after 8 weeks in culture. (**a**) Number of shoots per explant, (**b**) length of the main axillary cladode per explant, (**c**) fresh weight per explant, (**b**) dry weight per explant and (**e**) photo showing multiplication of cladode segment and cladode tip explants. NS = non-significant, * = significant at *p* ≤ 0.01.

The in vitro explant type significantly influenced the axillary cladode multiplication of *H. polyrhizus* in gelled media (Figure 1a–e). Cladode segments significantly produced a higher number of shoots and fresh and dry weights as compared with cladode tip explants. However, no significant (*p* = 0.2258) effect of explant type on the length of axillary cladodes was observed (Figure 1b). Previous studies highlighted that the cladode multiplication rate depends on the explant type and its position. Viñas et al. [12] studied the effect of position of the areoles on bud sprouting of the purple dragon fruit (*Hylocereus costaricensis*), indicating that the central region of the joint produced 82.5% shoots, in contrast to 61.9% and 67.7% from the apical and basal sections, respectively. Similarly, Dabekaussen et al. [30] recommended the utilization of the central section of *Sulcorebutia alba* as the basal and distal sections of sprouted irregular buds. These varied responses have been proposed to be due to the distribution of endogenous hormones within the stem [31]. In the present study, we thought that the low number of cladodes produced by the tip explants could possibly be due to the apical dominance that prevented sprouting of *H. polyrhizus* axillary cladodes. However, noticeably, both explant types produced axillary cladodes only at the proximal areoles attached to the medium. Therefore, the effects of the liquid culture system on axillary cladode multiplication of *H. polyrhizus* were investigated.

**Figure 2:**
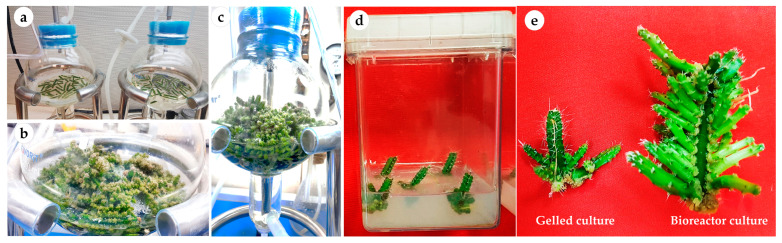
Multiplication and growth of *H. polyrhizus* in gelled culture versus bioreactor culture after 8 weeks. (**a**) Continuous immersion bioreactors at zero day of culture; (**b**,**c**) axillary cladode growth after 4 and 8 weeks in liquid culture; (**d**) axillary cladode growth after 8 weeks in gelled culture and (**e**) axillary cladode multiplication in gelled versus bioreactor cultures after 8 weeks.

The number of axillary cladodes, fresh and dry weights and length of the main axillary cladode were also significantly (*p* < 0.0001) influenced by the culture type (i.e., continuous immersion bioreactors and gelled culture) (Table 1; Figure 2). However, the culture type did not influence the total chlorophyll content. Explants of *H. polyrhizus* grown in liquid cultures produced a higher number of cladodes (42.8 and 45.9 with net and without net, respectively) than were produced in gelled culture (6.7 cladodes per explant). Liquid cultures using bioreactors resulted in cladodes with a higher fresh weight and length than those grown via gelled culture. Both immersion systems (with net or without net) were useful in terms of plant growth and multiplication, which is interesting for mass production of pitaya plantlets. Although no significant difference was obtained between the two immersion liquid cultures, continuous immersion without a net facilitated easy handling and harvesting of cladodes as compared with continuous immersion with a net.

The areoles contain meristematic tissues that can be activated to produce axillary buds. Gelled cultures resulted in low activation of areoles and cladode multiplication of *Hylocereus* species according to the literature [9,11,12,15,16]. Mohamed-Yassen [10] reported a maximum number of eight cladodes per decapitated explant of *H. undatus* cultured on MS medium containing 0.5 μM thidiazuron (TDZ) and 0.5 μM NAA. Similar findings for cladode multiplication of *H. undatus* with a maximum of 5, 7.2 and 8.2 cladodes per explant were reported by Viñas et al. [12], Hua et al. [11] and Fan et al. [9], respectively. For *H. polyrhizus,* Qin et al. [16] reported that the highest multiplication of *H. polyrhizus* was 6.4 adventitious cladodes per explant after 4 weeks in culture on MS medium fortified with 5.5 mg L^−1^ BA and 0.1 mg L^−1^ naphthalene acetic acid (NAA). In another study, the highest multiplication *H. polyrhizus* on MS medium supplemented with 3 mg L^−1^ BA was 3.67 ± 0.67 cladodes per explant [15]. Liquid medium is known to enhance nutrient uptake and encourage growth and proliferation in vitro. Additionally, aeration and mixing with oxygen are optimal for plant cultures in a bioreactor system [32,33]. Studies on cacti and succulent species in liquid/bioreactor cultures are few. A previous report indicated that *Euphorbia milli* microshoots exhibited superior growth and multiplication in continuous immersion bioreactor culture (+net; partial immersion of explants) than temporary immersion or gelled cultures [34,35]. Recently, Bello-Bello et al. [18] highlighted the efficiency of a temporary immersion system for micropropagation of *Hylocereus undatus* as compared with semi-solid cultures. The authors indicated that a temporary 2 min immersion every 4 h of 15 explants produced the highest number of multiple shoots (10.7 cladodes per explant) while semi-solid culture produced 5.1 cladodes per explant. However, no significant difference was recorded for cladode length in the cultures. Conversely, liquid culture systems are often associated with growth abnormalities and physiological disorders such as hyperhydricity, that involves accumulation of apoplastic water within the tissues, leading to eventual death of propagules [36,37] or inability to survive ex vitro hardening. In the present study, no symptoms of hyperhydricity were observed. Our findings encourage the utilization of bioreactor culture systems for mass propagation of other *Hylocereus* species. 

**Figure 3:**
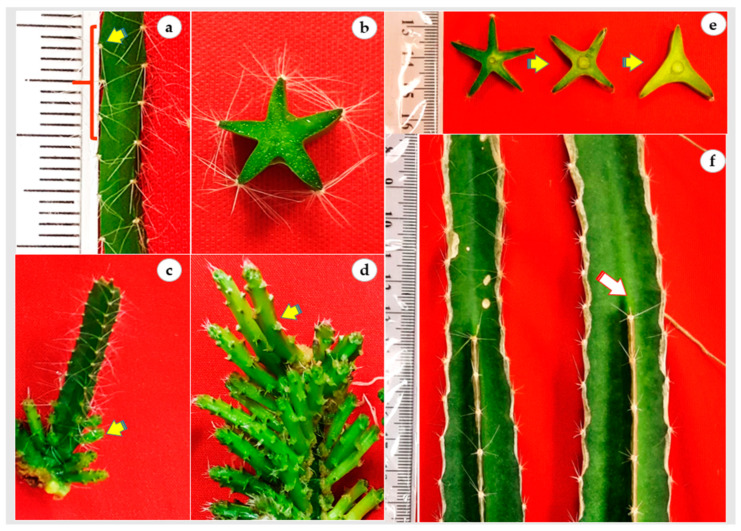
In vitro regeneration and development of *H. polyrhizus* axillary cladode. (**a**) Distribution and distance among areoles (≅3 mm; arrow) along the in vitro cladode; (**b**) transverse section of in vitro cladode showing five sides; (**c**; arrow) axillary cladode regeneration at the explant proximal areoles in gelled culture; (**d**) regeneration of both axillary cladode and secondary axillary cladode (arrow) from the whole areoles in air-lift bioreactor cultures; (**e**,**f**) reduction in number of cladode sides (arrow showing dead end) from 5 to 3 in mature plants.

In vitro cladode of *H. polyrhizus* had five sides and the distance between areoles was nearly 3 mm (Figure 3a,b). Thus, an in vitro cladode explant (cladode segments 3 cm in length) possessed nearly 45 areoles that could differentiate into 45 axillary cladodes. In the present study, all areoles were activated and produced axillary cladodes in the continuous immersion bioreactor cultures whereas only proximal areoles were induced in gelled culture (Figure 3c,d). Additionally, it is noted that all axillary cladodes in bioreactors had a similar length, probably due to the homogenous availability of hormones and nutrients, while the length acropetally decreased in gelled culture. It is also noted that the number of *H. polyrhizus* cladode sides were reduced from five sides to three sides during maturation to the adult stage (Figure 3e,f, arrow). Since *H. polyrhizus* is grown in arid environments that are characterized by shortages of water, low rainfall or drought conditions, the reduction of cladode sides could be a survival strategy through reducing the plant surface.

In the present study, in vitro rooting of *H. polyrhizus* axillary cladodes was achieved easily within 4 weeks with 100% rooting following their culture on MS medium without PGRs. Similarly, MS medium without PGRs was utilized for in vitro rooting of *H. undatus* [10]. Auxin supplementation to the in vitro rooting medium may reduce rooting percentage and retard rooting due to callus formation at the cladode base. Inclusion of 0.3 mg L^−1^ indole butyric acid (IBA) and 0.5 mg L^−1^ NAA in the rooting medium resulted in 92% rooting of *H. polyrhizus* [16]. 

### 2.2. Effect of AMF on Acclimatization of Micropropagated H. polyrhizus Plantlets

**Figure 4:**
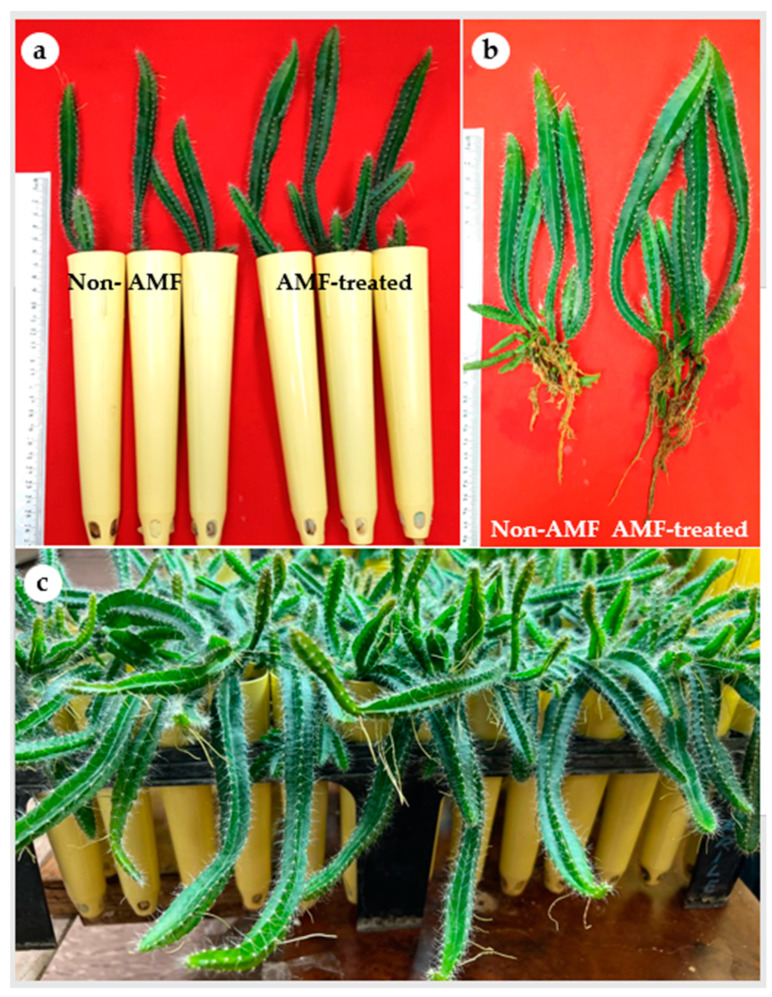
Influence of arbuscular mycorrhizal fungi on growth of *H. polyrhizus* after 8 weeks of acclimatization. (**a**) Non-AMF plantlets and AMF-treated plantlets grown in pots filled with sterile sand and soil (1:1; *v/v*), (**b**) root growth of non-AMF plantlets and AMF-treated plantlets and (**c**) micropropagated plantlets after 12 weeks of acclimatization.

In the present study, all *H. polyrhizus* plantlets showed 100% survival following their acclimatization independent of the mycorrhizal treatment. It has been noticed that cacti and succulent plant species can be rehydrated owing to their well-developed cuticle [38]. Previous studies highlighted that micropropagated *Hylocereus* had a high survival percentage during acclimatization, i.e., 98–100% for *H. undatus* [18], 96% for *H. costaricensis* [12] and 100% for *H. polyrhizus* [16]. Similarly, a 100% survival rate of micropropagated *E. millii*, a succulent plant species of Euphorbiaceae, was reported [34]. Microscopic investigation of the mycorrhizal colonization status of *H. polyrhizus* plantlets indicated the presence of all predicted AMF structures (mycelium, vesicles, arbuscules and spores) in the roots (Supplementary Figure S1a–d). The analysis of the mycorrhizal colonization showed the total colonization percentage of mycelium was 64.44%. The total spore count was also recorded as 75/100 g soil (Table 2). AMF-treated *H. polyrhizus* plantlets exhibited significantly higher values of length of the main cladode, length of the main root, fresh and dry biomass, content of chlorophyll a and total carotenoids compared to non-treated plantlets (Table 2; Figure 4a,b). However, the number of cladodes per plantlet and the quantity of chlorophyll b were similar in AMF-treated and control plantlets (Table 2). 

AMF have been successfully applied to improve vegetative growth and survival of several micropropagated plant species in ex vitro conditions. However, the degree of fungus–plant compatibility is genetically controlled by the symbionts [39]. Using AMF inoculum for micropropagated *Gerbera* sp. resulted in higher fresh and dry biomass and root length compared to the non-inoculated plantlets [40]. Similarly, micropropagated *Etlingera elatior* plantlets inoculated with AMF had a superior growth performance and increased plant survival by 50% compared to non-AMF plantlets [41]. Micropropagated *Anthurium andraeanum* plantlets inoculated with AMF exhibited better vegetative growth during acclimatization compared to the non-AMF plantlets [42]. Inoculation of micropropagated *Vitis vinifera* plantlets with different AMF strains significantly had enhanced chlorophyll and carotenoid content in the leaves and all the AMF treatments either singly or in combination were significantly superior to non-inoculated plantlets [43]. High chlorophyll content was also reported in AMF-treated plantlets of *Capsicum annuum* [29] and *Vitis vinifera* [44]. In view of the previous literature, AMF treatment not only facilitates the production of high-quality micropropagated plantlets and better adaptation to ex vitro environments but could also reduce the production costs during the acclimatization. It has been reported that AMF treatment of micropropagated *Syngonium podophyllum* and *Draceana* sp reduced the acclimatization period by fifteen days [45].

In the present study, AMF proved a viable biotechnological tool for improved growth of *H. polyrhizus* plantlets during the acclimatization stage. Our results indicated that AMF-treated *H. polyrhizus* plantlets had higher content of leaf pigments as compared with non-AMF plantlets. This increment in chlorophyll content is associated with high photosynthetic capacity resulting in vigorous vegetative growth during acclimatization (Figure 4c). Additionally, the increased carotenoid content in AMF plantlets implies their greater ability to survive the stressful conditions during acclimatization as compared with non-AMF plantlets. Carotenoids act as accessory light-harvesting pigments. They also perform photoprotective roles by quenching triplet state chlorophyll molecules and scavenging singlet oxygen and other toxic oxygen species formed within the chloroplast [46]. Overall, the findings of the present study demonstrate the beneficial effects of AMF for enhanced growth of micropropagated *H. polyrhizus*. Additionally, taking into consideration the slow growth nature of *H. polyrhizus,* AMF inoculation shows potential importance for large-scale micropropagation of this fruit crop. 

## 3. Materials and Methods

### 3.1. Plant Material and Establishment of In Vitro Culture

**Figure 5:**
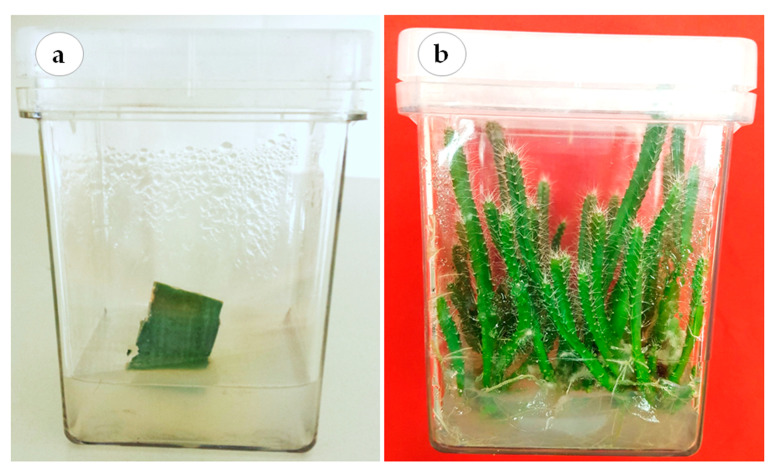
Establishment of *H. polyrhizus* in vitro culture. (**a**) Aseptic cladode segment grown on hormone-free MS medium and (**b**) in vitro axillary cladodes, used as plant materials for conducting the experiments.

This study was conducted at the laboratory of plant tissue culture, College of Food and Agricultural Sciences, King Saud University. The cladode segments of red dragon fruit (*H. polyrhizus* (F.A.C. Weber) Britton & Rose) were surface disinfected in 70% (*v/v*) ethanol for 30 s followed by 20% (*v/v*) of 5.25% sodium hypochlorite solution for 20 min. The explants were washed 4–5 times with sterile distilled water prior to their culture onto hormone-free Murashige and Skoog’s (MS) medium [47] (Figure 5a). The medium was supplemented with 30 g L^−1^ sucrose and solidified using 8.0 g L^−1^ agar agar (Dephyte). The pH of the medium was adjusted to 5.8 before autoclaving at 121 °C and 118 kPa pressure for 15 min. The cultures were incubated at 23 °C ± 2 °C air temperature and 70 μmol·m^−2^·s^−1^ photosynthetic photon flux density (PPFD) provided by cool white fluorescent tubes under a 16:8 h (light: dark) photoperiod. The axillary cladodes were routinely sub-cultured until enough plant material was available to conduct the experiments in this study (Figure 5b).

### 3.2. Effects of Explant Type on Axillary Cladode Multiplication of H. polyrhizus in Gelled Culture

Two types of explants were tested: Cladode segments and tips (3 cm length) were cultured in Magenta GA-7 culture vessels (77 × 77 × 97 mm; Sigma Chemical Co., St. Louis, MO, USA) that contained MS medium supplemented with 30 g L^−1^ of sucrose, 1 mg L^−1^ of BAP 6-benzylaminopurine and 0.1 mg L^−1^ of NAA naphthalene acetic acid and solidified with 0.8% agar agar. The pH of the medium was adjusted to 5.8 prior to autoclaving at 121 °C and 1.2 kg cm^−2^ pressure for 20 min. The environmental conditions of the growth chamber were adjusted to 23 °C ± 2 °C and 70 μmol m^−2^ s^−1^ PPFD for a 16 h photoperiod using cool white fluorescent lamps. The growth and cladode multiplication characteristics, i.e., the number of cladodes per explant, cladode length and fresh and dry weights, were compared for both explant types after 8 weeks in culture.

### 3.3. Effects of Gelled and Liquid Bioreactor Culture on Axillary Cladode Multiplication of H. polyrhizus

**Figure 6:**
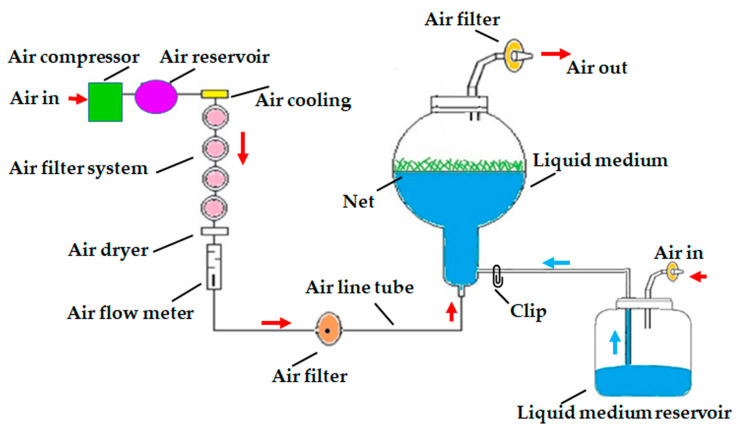
Schematic diagram of air-lift continuous immersion liquid culture system utilized for axillary cladode proliferation of *H. polyrhizus* using cladode segments. Red arrow = supplement of air, blue arrow = supplement of liquid medium.

Two treatments of an air-lift bioreactor liquid culture system (continuous immersion with a net or without a net; PLT Scientific SDN BHD, Puchong, Selangor D.E., Malaysia), were tested for axillary cladode multiplication production in liquid media and compared with gelled culture. Cladode segments (40 explants per bioreactor) were transferred to a 3 L balloon-type bubble bioreactor with 1.2 L of MS liquid medium supplemented with 30 g L^−1^ of sucrose, 1 mg L^−1^ of BAP and 0.1 mg L^−−1^ of NAA. The pH of the medium was adjusted to 5.8 before autoclaving at 121 °C and 1.2 kg cm^−2^ pressure for 30 min. In the immersion-type bioreactor, a supporting plastic net was used to hold the plant material to avoid the complete submersion of explants in the liquid medium (Figure 6). The volume of air input was adjusted to 0.1 air volume/culture volume min^−1^ (vvm). All the bioreactors and culture vessels were maintained at 23 °C ± 2 °C and 70 μmol m^−2^ s^−1^ PPFD for a 16 h photoperiod using cool white fluorescent lamps. The number of axillary cladodes, length of the main axillary cladode, fresh weight, dry weight and total chlorophyll content were recorded after 8 weeks of culture from 15 randomly selected explants.

### 3.4. In Vitro Rooting, AMF Inoculation and Acclimatization of Micropropagated H. polyrhizus Plantlets

Proliferated axillary cladodes of *H. polyrhizus*, an easy to root species, were cultured on auxin-free MS medium for their in vitro rooting. After 4 weeks, the plantlets were gently removed from the gelled medium and cleaned using tap water. Two treatments—with or without AMF inoculation—were applied. The plantlets were transplanted into conical plastic pots (4 cm upper diameter; 1.7 cm bottom diameter; 21.5 cm in length) filled with a sterilized sand: soil (1:1) mixture and amended with 5% (*w/w*) AMF inoculum soil. The applied inoculum comprising AMF species *Gigaspora margarita* and *Gigaspora albida* with a density of 33.4 spores g^−1^ dry soil was acquired from the Rangeland Lab of the Plant Production Department, King Saud University. The non-AMF plantlets received the same dosage of autoclaved AMF inoculum. Thereafter, the potted plants were grown at 25 ± 2 °C, 50–60% RH and 100 µmol m^−2^ s^−1^ PPFD (16:8 h photoperiod under white fluorescent lamps) in a growth chamber with the pots covered with transparent polyethylene for the first 2 weeks. The plantlets were regularly irrigated with Hoagland nutrient solution without phosphorus. Plantlet growth, mycorrhizal condition/status and survival were evaluated 8 weeks after being transferred to the growth chamber. There were two treatments, with or without mycorrhizal inoculation. Each treatment had 30 replicates and each replicate was represented by a pot containing one micropropagated plantlet.

### 3.5. Symbiotic Development and AMF Spore Counts

To quantify the percentage of root colonization, fresh fine roots were carefully selected, stained and studied by following the methods of Phillips and Hayman [48] with some modifications according to Al-Qarawi et al. [49]. From the substrate of each treatment, the spores were isolated as per the methods of Gerdemann and Nicolson [50]. The total spore population in each treatment was calculated based on 100 g of dry soil [51].

### 3.6. Measurements of Leaf Pigments and Vegetative Parameters 

To measure chlorophyll a, b and total carotenoids, 0.5 g of cladodes from each treatment was extracted for 48 h in 80% cold acetone, and the absorbance was measured at 663.2, 646.8 and 470.0 nm, respectively. There were three replicates in each treatment and calculations were made following the method of Lichtenthaler [52]. Growth responses in terms of number of cladodes, length of the main cladode (cm), length of the main root (cm), fresh and dry weights per plantlet (g) were measured after 8 weeks of culture. The dry weight was recorded after oven-drying the samples for 48 h at 70 °C. All measurements were obtained from 15 randomly chosen plantlets. 

### 3.7. Experimental Design and Data Analysis

The experiments had a completely randomized design. The treatment effects were assessed statistically using ANOVA, unpaired *t*-test and Tukey’s multiple range test in the SAS program (Version 9.4; SAS Institute, Inc., Cary, NC, USA).

## 4. Conclusions

In conclusion, liquid culture activated *H. polyrhizus* areoles, resulting in a six-fold higher multiplication rate of axillary cladodes compared to gelled culture. An efficient cladode proliferation of *H. polyrhizus* was developed using an air-lift continuous immersion bioreactor. Inoculation of *H. polyrhizus* micropropagated plantlets with AMF (*G. margarita* and *G. albida*) enhanced the vegetative growth during acclimatization. The association of those two biotechnological tools facilitated large-scale micropropagation and the production of high-quality acclimatized *H. polyrhizus* plantlets in ex vitro conditions.

## Figures and Tables

**Table 1:** Effect of culture method on multiplication and growth of *H. polyrhizus* after 8 weeks of culture.

Culture Method	Number of Cladodes /Explant	Fresh Weight /Explant (g)	Dry Weight /Explant (g)	Length of the Main Axillary Cladode (cm)	Total Chlorophyll Content (mg g^−1^FW)
Gelled culture	6.7 b	0.872 b	0.120 b	1.76 b	0.255 a
Air-lift bioreactor (without net)	45.9 a	9.405 a	0.677 a	3.03 a	0.222 a
Air-lift bioreactor (with net)	42.8 a	7.358 a	0.598 a	3.11 a	0.253 a
*F*-value	84.26	22.38	43.46	19.80	0.51
*p*-value	<0.0001 *	<0.0001 *	<0.0001 *	<0.0001 *	0.6232 ^NS^

Values followed by the same letter in the same column are not significantly different at *p* ≤ 0.05 level, according to Tukey’s range test. NS = non-significant, * = significant at *p* ≤ 0.001.

**Table 2:** Vegetative growth characteristics, leaf pigments, total colonization percentage and AMF spore count of *H. polyrhizus* in response to arbuscular mycorrhizal fungi after 8 weeks of treatment.

Growth Parameters	Non-AMF	AMF-Treated	*p*-Value
Number of cladodes/plant	3.30 ± 0.236	4.33 ± 0.471	0.0533 ^NS^
Length of the main cladode (cm)	18.50 ± 0.408	24.83 ± 0.717	0.0001 *
Length of the main root (cm)	8.00 ± 0.540	12.17 ± 0.425	0.0005 *
Fresh weight/plantlet (g)	9.679 ± 0.056	14.107 ± 0.235	<0.0001 *
Dry weight/plantlet (g)	0.853 ± 0.009	1.073 ± 0.074	0.0130 *
Chlorophyll a (mg g^−1^FW)	0.499 ± 0.060	0.683 ± 0.091	0.0304 *
Chlorophyll b (mg g^−1^FW)	0.208 ± 0.009	0.198 ± 0.013	0.2314 ^NS^
Chlorophyll a+b (mg g^−1^FW)	0.707 ± 0.052	0.881 ± 0.090	0.0317 *
Carotenoids (mg g^−1^FW)	0.129 ± 0.018	0.240 ± 0.030	0.0018 *
Mycorrhizal colonization (%)	0	64.444 ± 2.222	0.000 *
AMF spore counts	0	75.667 ± 5.507	0.000 *

Data presented are means ± standard error. NS = non-significant and * = significant at 5% level according to unpaired *t*-test.

## Data Availability

All data are presented in the article.

## Supplementary Materials

The following supporting information can be downloaded at: https://www.mdpi.com/article/10.3390/plants12051037/s1, Figure S1: The perfect abundance of arbuscular mycorrhizal fungi colonization in the roots of micropropagated *Hylocereus polyrhizus* plantlets.
