# Supplementary material for: Promising Application of Automated Liquid Culture System and Arbuscular Mycorrhizal Fungi for Large-Scale Micropropagation of Red Dragon Fruit
Source: Plants (Basel). 2023 Feb 24;12(5):1037. doi: 10.3390/plants12051037 (PMC10005386; doi:10.3390/plants12051037)
Supplement: Supplementary file 1 [file plants-12-01037-s001.zip › plants-2181769-supplementary.pdf]

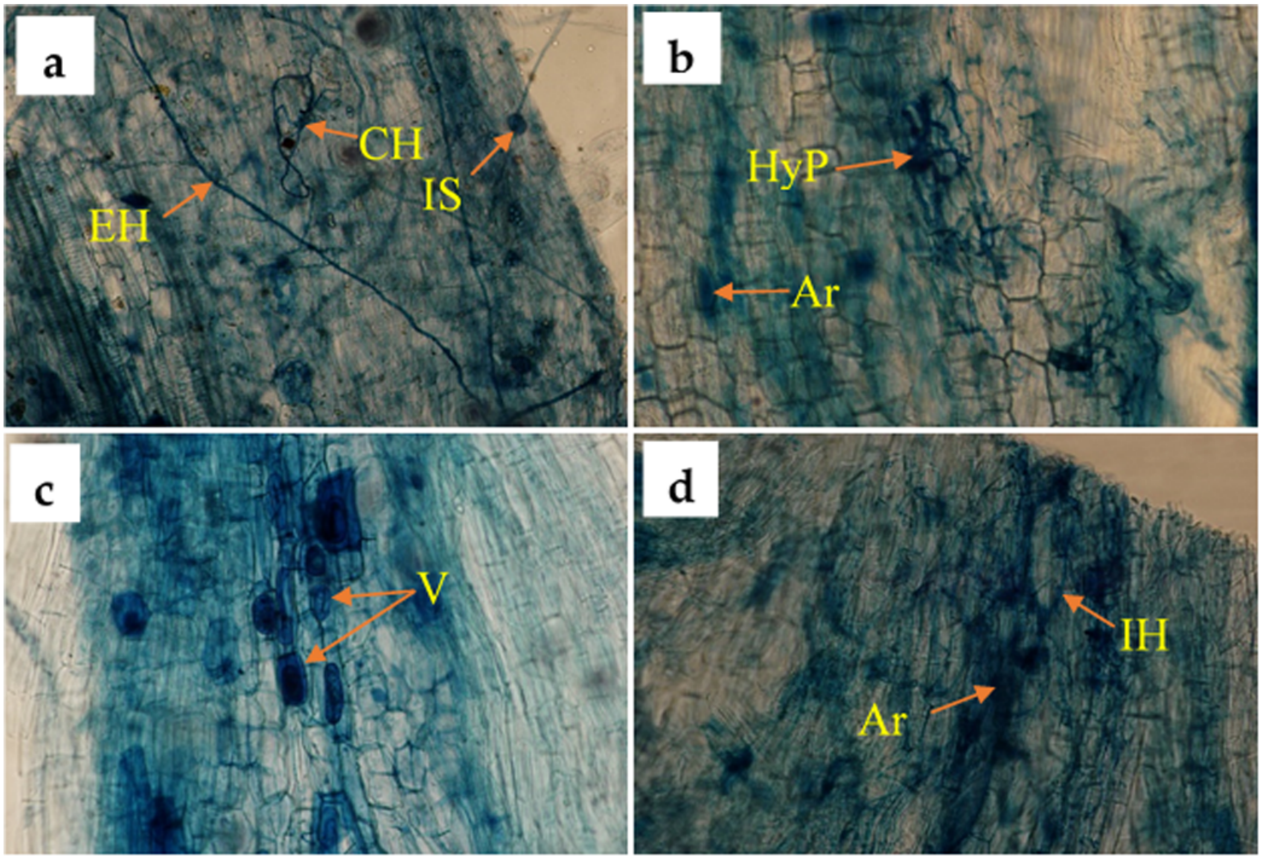

Supplementary Figure S1. The perfect abundance of arbuscular mycorrhizal fungi colonization in the roots of micropropagated *Hylocereus polyrhizus* plantlets. (a–d) the presence of hyphopodium (HyP) indicated AMF colonization, which later propagated and developed various structural forms. Intraradical spores (IS); External hyphae (EH); arbusculate hyphal coils (CH); Vesicles (V); Intercellular hyphae (IH) and Arbuscules (Ar).
